# Diagnostic challenges in high myopia: identification of sight-threatening complications and the role of artificial intelligence

**DOI:** 10.3389/fopht.2025.1727063

**Published:** 2026-01-15

**Authors:** Shiqi Zhang, Jiaqi Chen, Hongli Yang, Huiping Yuan

**Affiliations:** 1 Department of Ophthalmology, The Second Affiliated Hospital of Harbin Medical University, Harbin, China; 2 Devers Eye Institute, Legacy Health Research, Portland, OR, United States

**Keywords:** artificial intelligence (AI), complication diagnosis, high myopia, optical coherence tomography (OCT), pathologic myopia

## Abstract

High myopia (HM), defined as a spherical equivalent refractive error ≤ -5.00 or ≤ -6.00 diopters or axial length (AL) ≥ 26.0 mm, is a significant public health concern with a rapidly increasing prevalence, particularly in East Asia. Beyond impaired uncorrected vision, HM is associated with sight-threatening structural changes, including myopic maculopathy, choroidal neovascularization, retinal detachment, and glaucoma. The overlapping and atypical presentations of these complications pose considerable diagnostic challenges, often delaying intervention and complicating clinical management. This review synthesizes current knowledge on HM, emphasizing the spectrum of ocular complications and the multifaceted diagnostic dilemmas encountered. We have summarized the application of conventional and emerging diagnostic techniques—such as optical coherence tomography (OCT), ultra-widefield imaging, and fluorescein angiography in the diagnosis of high myopia and highlight the growing role of artificial intelligence (AI) and machine learning in enhancing diagnostic accuracy, particularly through the analysis of retinal images and OCT data. AI-based systems demonstrate high sensitivity and specificity in detecting HM-related pathologies, offering potential for large-scale screening and early intervention. Future directions include the development of integrated multimodal imaging platforms, genetic and metabolic biomarkers, and AI-driven predictive models to support personalized management strategies. This comprehensive overview underscores the need for advanced, accessible diagnostic tools to alleviate the burden associated with high myopia.

## 1 Introduction

Myopia, the most prevalent form of refractive error globally, presents a significant and escalating public health challenge (1). High myopia (HM), generally defined as a refractive error with a spherical equivalent (SE) of ≤ -5.00 diopters (D) or ≤ -6.00 D (2–4), represents the more severe end of the myopic spectrum. The prevalence of high myopia has increased rapidly, particularly in the Asian population (5). While the refractive error compromises uncorrected distance vision, the principal implication of high myopia lies in its associated pathological changes, which carry a significant risk of sight-threatening conditions. It is often progressive, structural alterations within the eye referred to as pathological or degenerative myopia. These complications include myopic macular degeneration, choroidal neovascularization, retinal detachment, macular schisis, chorioretinal atrophy, and glaucoma, among others (6). The diverse and complex nature of these complications, which frequently present with overlapping or atypical clinical features, creates significant diagnostic challenges for clinicians. This inherent difficulty in accurately identifying and differentiating HM-related pathologies hinders timely intervention and comprehensive management. This review aims to synthesize current knowledge on high myopia, examining its ocular complications, and specifically addresses the multifaceted diagnostic dilemmas encountered in its evaluation and identification.

## 2 High myopia

### 2.1 Definition

High myopia, a prevalent and potentially vision-threatening condition, has drawn increasing concern due to its growing global incidence. Diagnosis is typically based on refractive error measurements, as described earlier, when ocular accommodation is relaxed (4). However, this refractive-based definition has limitations, as it fails to capture the full spectrum of structural and pathological alterations in the eye. AL plays a critical role in diagnosis as well. For example, individuals with flat corneas may exhibit ocular changes characteristic of high myopia due to excessive axial elongation, even if their refractive error does not meet conventional diagnostic thresholds. Therefore, high myopia is also defined in some contexts as an axial length (AL) ≥ 26.0 mm (4, 7–9).

### 2.2 Epidemiology

Epidemiologic studies indicate a rising global prevalence of myopia, with particularly marked increases in East Asian countries. Over the past 10 to 15 years, the rate of high myopia has risen from below 10% to approximately 10–20% in certain East Asian populations (10). In contrast, the prevalence of high myopia in non-Asian regions remains relatively low. A meta-analysis of data from the European Eye Epidemiology (E3) Consortium, which included refractive error measurements from 61,946 individuals, reported an age-standardized prevalence of high myopia of 2.7% in Europe (11), underscoring the substantial burden of myopia in this region. Reported rates of high myopia vary widely across other populations: 0.3% in Denmark (mean age 19.3 years), 1.9% among 17-year-olds in Australia, 2.0% in men and 2.3% in women aged 16–22 years in Israel, and 3.92% in the United States (10). Among African schoolchildren, the prevalence of myopia is lower than in other parts of the world (12).

Environmental and lifestyle changes have significantly contributed to the increasing prevalence of myopia and high myopia worldwide (13). The growing incidence of early-onset myopia—defined by the International Myopia Institute (IMI) as myopia where the refractive error exceeds what is considered typical for the child’s age group—extends the period of refractive progression, as affected children often continue to progress until their mid-20s (14). This prolonged window of progression elevates the risk of developing high myopia. The rising trend of early-onset myopia among those who eventually develop high myopia portends a worsening epidemic in the coming decades, given that a substantial proportion of these individuals are likely to progress to pathologic myopia (15).

### 2.3 Genetics

Refractive error has a highly heritable trait, with genetic factors representing the primary determinant of its variation within populations. Genetically, myopia manifests in two distinct forms: “common/polygenic” myopia, driven by the cumulative effect of numerous common genetic variants that collectively elevate myopia risk, and monogenic syndromic myopia, which follows Mendelian inheritance patterns (16). The former is modifiable by environmental and lifestyle factors, whereas the latter arises from a single pathogenic variant and is frequently associated with systemic features. *OPN1LW*, *COL2A1*, *COL11A1*, and *P3H2* are associated with Mendelian or syndromic myopia. For example, Cohen syndrome—an autosomal recessive disorder characterized by progressive retinochoroidal dystrophy, high myopia, and other manifestations—results from biallelic pathogenic variants in *VPS13B*, with diagnosis confirmed through clinical assessment and genetic testing (17).

Current evidence has identified several genes responsible for Mendelian forms of non-syndromic high myopia. *ARR3*, which represents a significant monogenic factor for isolated early-onset high myopia in certain cohorts, demonstrates an X-linked inheritance pattern with female predominance (18). *KDELR3*, identified through whole-exome association studies of Chinese individuals with high myopia (19). Despite the high heritability of myopia and the consensus from epidemiological studies identifying family history as its predominant risk factor, not all cases of high myopia can be fully predicted by genetic factors alone. Genetic testing may offer clinical utility for high-risk groups (e.g., individuals with a positive family history), whereas its applicability in population-wide screening remains limited.

## 3 Pathologic myopia

Pathologic myopia (PM) is defined as a sight-threatening condition characterized by degenerative changes in the posterior segment secondary to excessive axial elongation in highly myopic eyes (20). It represents an advanced stage of high myopia and is frequently confused with high myopia itself (13). It is critical to note that not all eyes with high myopia will progress to this pathologic state. The hallmark manifestations include posterior staphyloma, myopic maculopathy (such as diffuse or patchy chorioretinal atrophy, lacquer cracks, and macular atrophy), and optic neuropathy (13). These structural alterations are the primary drivers of irreversible visual impairment in affected individuals.

The pathological and physiological alterations associated with PM constitute the foundation for its clinical diagnosis. PM is driven primarily by excessive axial elongation—a process often influenced by environmental factors (3). This elongation places biomechanical stress on ocular tissues, leading to pathological alterations such as scleral thinning and compromised biomechanics, which contribute to posterior staphyloma formation (21). As a hallmark of pathologic myopia, posterior staphyloma exerts traction on the retina and choroid, ultimately resulting in various maculopathies.

The development and severity of these pathologic changes are influenced by several factors beyond a simple refractive error or AL measurement. A key determinant is age, as the prevalence of pathologic myopia is significantly higher in middle-aged and elderly populations (0.9%-3.1%) compared to children and adolescents (<0.2%) (22), indicating a progressive, time-dependent deterioration in high myopia. The choroid also plays a crucial role in the pathophysiology of pathological myopia. Choroidal thickness (ChT) decreases significantly with the increase of myopia and AL (23), which is a well-documented risk factor for myopic maculopathy (24). For instance, specific cut-off values for macular ChT have been proposed to classify the severity of chorioretinal atrophy, underscoring its prognostic value (25).

In addition to mechanical stretching and choroidal thinning, other pathways contribute to the complex pathophysiology. Oxidative stress has also been implicated in the pathophysiology of high myopia (26, 27). Hypoxia in the eyes of long ocular axis leads to oxidative damage, and this can disrupt the neuromodulation of eye growth and directly damage the retina, vitreous, and lens, contributing to complications like retinopathy, retinal detachment, and cataract formation (28–31). Thus, the progression to pathologic myopia is a multifactorial process involving biomechanical, vascular, cellular, and molecular insults.

Numerous susceptibility genes associated with myopia and high myopia have not been found to correlate with myopic macular neovascularization (MNV) (32, 33). However, a genome-wide association study (GWAS) focusing on myopic macular degeneration within the Japanese population identified CCDC102B as a susceptibility gene for this condition, revealing no significant association with axial length of the eye (34). The identification of CCDC102B suggests that genetic diagnostics could play a role in aiding the prevention of myopic macular degeneration.

## 4 Diagnosis of high myopia complications

### 4.1 Diagnostic technique

Diagnosing HM and PM requires a comprehensive approach that combines various diagnostic techniques to accurately assess the condition and its associated complications. Fundus photography is another important tool. It can provide a wide-field view of the retina, allowing for the detection of lesions such as lacquer cracks, chorioretinal atrophy, and Fuchs spot. Novel imaging modalities, such as ultra-widefield (UWF) imaging with a field of view ranging from 100 to 200 degrees, can capture retinal lesions missed by traditional color fundus photography, leading to improved screening accuracy and early detection (35). However, fundus photography alone may not be sufficient to detect some subtle changes, especially in the early stages of the disease.

Optical coherence tomography (OCT) is a cornerstone in the diagnosis of several complications. It can provide high-resolution cross-sectional images of the retina, choroid, and sclera, allowing for the detection of characteristic features such as myopic foveoschisis, posterior staphyloma, and choroidal thinning (36). New OCT technologies such as EDI-OCT and SS-OCT have led to greater insight into the pathophysiology of HM and its complications (37). Optical coherence tomography angiography (OCTA) enables the visualization of the retinal microvascular system without the need for contrast agents, offering detailed imaging of vascular networks across various retinal depths (38). This capability positions OCTA as a highly effective diagnostic tool for the screening of complications associated with high myopia.

Fluorescein angiography (FA) has better repeatability and reproducibility to make the diagnosis of myopic CNV (39). It enables the understanding of the physio-pathological process in eyes with myopic changes and is useful in detecting the presence and type of myopic CNV. FA can also help in differentiating CNV from other macular lesions. However, its invasive nature and potential side effects limit its routine use. Nowadays, OCTA can serve as a non-invasive alternative for certain diagnostic functions traditionally performed by FA (40). Video color-enhanced OCTA may help in diagnosing mMNV better than static OCTA (diagnostic rate 95% to 77%) (41).

In some cases, other inspection methods such as electroretinography (ERG) may be used, especially when there are concerns about retinal function. For example, in patients with Knobloch syndrome, which is associated with high myopia and retinal detachment, ERG can reveal cone-rod pattern dysfunction, providing valuable information about the underlying retinal pathology (42). To sum up, multimodal imaging plays a crucial role in the precise evaluation of pathologic myopia lesions (43).

### 4.2 Diagnosis in myopic maculopathy

Myopic maculopathy is a common complication of high myopia, and accurate diagnosis is essential for appropriate management. However, the global shortage of subspecialist-trained myopia experts and the diagnostic challenges faced by general eye care providers—such as optometrists and general ophthalmologists—highlight the need for standardized tools (20). The International Photographic Classification and Grading System for myopic maculopathy was developed to standardize the diagnosis and grading criteria (44). This system defines five categories of myopic maculopathy, including no myopic retinal degenerative lesion, tessellated fundus, diffuse chorioretinal atrophy, patchy chorioretinal atrophy, and macular atrophy. It also accounts for additional features like lacquer cracks, myopic choroidal neovascularization, and Fuchs spot, based on the grade of three variables: atrophy (A), traction (T), and neovascularization (N) (44). With established reliability and high reproducibility, this classification system enhances diagnostic consistency and improves communication across clinical and research settings (45).

OCT serves as the primary diagnostic tool for myopic maculopathy. It can detect various macular changes, such as myopic foveoschisis, macular hole, and choroidal neovascularization (CNV) (46). HM eyes have the larger foveal avascular zone (FAZ) and reduced perifoveal vessel density (VD) in both layers and less deep total VD (47). In cases of myopic foveoschisis, OCT clearly demonstrates splitting of the neural retina into a thicker inner layer and a thinner outer layer, and also allows for monitoring disease progression. For CNV, OCT reveals characteristic features including hyperreflective subretinal material, intraretinal or subretinal fluid, and retinal thickening. Through the integration of a multimodal imaging approach, including OCT, OCTA, FA, and ICGA, along with accurate demographic and clinical assessment, it becomes possible to effectively differentiate mCNV from similar yet heterogeneous entities (43, 48).

Emerging molecular diagnostic approaches offer promising avenues for predicting and identifying early myopic macular degeneration through high-throughput methods. Some plasma metabolic features, such as sebacic acid, have demonstrated diagnostic potential, with AUC values reaching 0.874 and 0.889 (30). Furthermore, Fan et al. identified 11 potential biomarkers correlated with the severity of atrophic myopic macular degeneration (AMM) (49). These findings indicate that blood-based biomarkers could play a future role in the diagnosis and monitoring of myopic maculopathy, although further validation is required to establish their clinical utility.

### 4.3 Glaucoma in myopia eyes

Myopia, especially high myopia, is associated with an increased risk of glaucoma. The pathophysiology underlying this association involves several structural and biomechanical changes in the eye. One key aspect is the change in the lamina cribrosa, which has been found to be thinner in myopic eyes, potentially increasing the susceptibility of the optic nerve to intraocular pressure (IOP) (50, 51). Additionally, myopic eyes often present with an enlarged optic disc, tilted disc, and peripapillary atrophy, which can resemble glaucomatous changes and complicate accurate diagnosis (52). Choroidal and retinal nerve fiber layer (RNFL) thickness has also been shown to be associated with myopia (23). These overlapping structural characteristics between myopic optic neuropathy and glaucomatous damage make the diagnosis of glaucoma particularly challenging in myopic individuals.

OCT enables three-dimensional, real-time visualization and quantitative assessment of the optic nerve head morphology, making it a valuable tool for detecting and monitoring glaucoma. As a result, it has been widely adopted in clinical practice (53). When compared to early glaucoma patients with high myopia, highly myopic eyes exhibit distinct patterns of retinal RNFL thickness (54). Specifically, compared to normal eyes, highly myopic eyes have a greater ability to detect issues using temporal quadrant cpRNFL thickness (higher AUC), but a reduced ability in the superior and inferior quadrants. These differences make it difficult to differentiate between glaucomatous damage and myopia-related structural changes based on RNFL thickness alone.

Researchers have investigated various markers to improve glaucoma diagnosis in myopic patients. A key anatomical reference is the Bruch’s membrane opening (BMO), which lies at the parapapillary end of Bruch’s membrane. The BMO-minimum rim width (BMO-MRW), measured as the shortest distance from the BMO to the internal limiting membrane (ILM), has proven valuable for early glaucoma detection, demonstrating higher sensitivity at 95% specificity compared to traditional RNFL thickness measurements (55–57).To enhance diagnostic accuracy in myopic eyes, RNFL thickness—particularly in the inferotemporal quadrant—can be analyzed alongside BMO-MRW around the optic disc. These structures are aligned using the Anatomical Positioning System (APS) software based on the fovea-to-BMO-center axis (57). This combined approach improves differentiation between glaucomatous and myopic changes. Moreover, relative to conventional RNFL parameters, BMO-MRW exhibits comparable or superior sensitivity, higher specificity, a reduced false positive rate, and better sectoral diagnostic performance in myopic populations (58–61). Thus, BMO-MRW represents a crucial diagnostic parameter, particularly in the context of high myopia.

Sectoral peripapillary retinal nerve fiber layer thickness (pRNFLT) and ganglion cell inner plexiform layer thickness (GCIPLT) have also demonstrated potential for improving glaucoma detection in highly myopic eyes (62). Beyond these retinal layers, structural changes in the lamina cribrosa (LC) offer further diagnostic insight. LC defects are significantly more common in myopic eyes with glaucoma than in those without (63). In particular, disinsertion-type LC defects(defined as a posteriorly displaced laminar insertion with a downward slope at the far periphery of the LC toward the neural canal wall) have been correlated with myopia-related parameters such as AL and the γ-zone peripapillary atrophy (PPA), as well as with the presence of glaucoma itself (64). Closely related to these structural alterations is peripapillary atrophy (PPA), which is frequently observed in both myopic and glaucomatous eyes. Notably, highly myopic eyes exhibiting larger γ-zone PPA (γPPA) warrant special attention, as this feature may signal an increased risk of early glaucomatous visual impairment (65).

Additionally, wide-field optical coherence tomography angiography (WF-OCTA) has emerged as a promising tool for diagnosing glaucoma in highly myopic patients. WF-OCTA enables detailed evaluation of peripapillary and macular superficial vascular density, which often shows alterations in glaucomatous eyes with high myopia (66). This modality has demonstrated high diagnostic performance, with reported sensitivity and specificity of 87.28% and 86.94%, respectively, for detecting glaucoma in highly myopic eyes. Notably, while the diagnostic efficacy of peripapillary perfusion density varies, macular perfusion density maintains consistent diagnostic accuracy in both high myopia and non-high myopia patients. Consequently, macular imaging using OCTA and OCT plays an essential role in the early diagnosis of glaucoma among individuals with high myopia (67, 68).

Visual field testing remains essential for glaucoma diagnosis; however, its accuracy in myopic patients is often compromised by structural ocular changes. To address these challenges, adapted testing strategies have been developed. For instance, the 10–2 protocol, which focuses on the central visual field, may improve the detection of early glaucomatous damage, as myopic eyes frequently exhibit central defects (69). Additionally, high myopia can induce a myopic shift in visual field results, blurring the distinction between true glaucomatous loss and artifacts caused by refractive error. One proposed approach is to adjust testing parameters according to refractive status. Emerging technologies such as objective perimetry, which assesses regional delays and inter-eye asymmetry, hold promise for improving diagnostic precision in myopic patients (70). Further research is warranted to refine perimetric strategies tailored to this population.

### 4.4 Retinal detachment in high myopia

Retinal detachment is a serious complication of high myopia, making early identification and appropriate management critically important. Currently, the identification of retinal detachment in high myopia often relies on a combination of patient symptoms and clinical examinations. Common symptoms include sudden onset of floaters, flashes of light, or a curtain-like shadow in the visual field. A study found that subjective visual reduction was strongly associated with retinal pathology in patients presenting with acute monocular floaters or flashes, showing a likelihood ratio of 7.9 (95% CI 5.2–12.1) (71). This indicates the importance of prompt ophthalmologic evaluation in such patients to rule out retinal detachment.

Retinal breaks and degenerative areas associated with high myopia constitute significant risk factors for retinal detachment, especially in the peripheral retina. Traditional diagnostic methods, such as fundus examination after dilated pupils, allow direct visualization of retinal breaks, tears, or detachments, yet face limitations in large-scale screening of the peripheral retina. Indirect ophthalmoscopy is prone to missed or misdiagnosed lesions—particularly in eyes with refractive media opacities—due to its relatively low magnification, inverted image presentation, and steep learning curve (72). Similarly, three-mirror contact lens examination offers a wide field of view and a strong stereoscopic effect, but requires contact with the cornea, often causing patient discomfort and poor cooperation. Moreover, they are not capable of recording the examination in pictures or videos (73). These challenges are compounded in highly myopic eyes, where structural changes such as posterior staphyloma and other maculopathies may elevate detachment risk and further reduce examination accuracy.

The advent of wide-field fundus imaging has introduced a more effective approach (74). For instance, the 200-degree ultra-wide-angle laser ophthalmoscope (Datonna) detected peripheral retinal lesions at a positive rate of 15.50%, comparable to that of three-mirror contact lenses (75). Furthermore, modern imaging systems such as Opel Panorama 200 and Zeiss Clarus 500 demonstrated high and consistent sensitivity in identifying retinal breaks—including those in the far temporal periphery—and offer improved performance in eyes with refractive media opacities (76).

Parallelly, the introduction of OCT has transformed the clinical diagnosis approach to retinal detachment by delivering high-resolution cross-sectional images of retinal layers. This capability allows precise assessment of detachment type, extent, and macular involvement, proving particularly valuable in cases with limited fundus visibility (77). Additionally, the advent of advanced OCT modalities, including SD-OCT, SS-OCT, OCT-A, adaptive optics OCT, and en face OCT, not only enhances diagnostic precision but also contributes to a more individualized treatment approach (78). Biomarkers—such as the integrity of the EZ, the presence of ORCS, and measurements of retinal detachment height—have markedly improved our ability to predict postoperative visual outcomes (78).

Beyond the structural and vascular biomarkers provided by OCT, other indicators have shown promise as potential screening tools for retinal detachment. For instance, hemodynamic parameters measured by carotid ultrasonography (CUS) have been associated with an elevated risk of retinal detachment (79). On the genetic level, GWAS have identified three significant single-nucleotide polymorphisms (SNPs) that are highly expressed at the retinal detachment border (79). Furthermore, for myopic eyes, fundus refraction offset continues to emerge as a viable personalized biomarker for assessing the risk of retinal breaks or detachment (80). Therefore, integrating multiple imaging modalities is essential for the early diagnosis and risk stratification of retinal detachment in patients with high myopia.

## 5 Emerging technologies

Emerging technologies are revolutionizing the detection and management of high myopia and its related complications. A particularly promising advancement lies in the application of artificial intelligence (AI) and machine learning (ML). Recent studies have shown a clear shift from traditional machine learning toward deep learning techniques in the diagnosis of myopia (81). AI-based systems demonstrate a strong capacity to analyze large volumes of ophthalmic data to facilitate the identification of high myopia and its complications, including fundus photographs, optical coherence tomography (OCT) scans, and other clinical parameters. Currently, AI has established itself as a powerful tool in diagnosing myopic maculopathy and glaucoma. Nevertheless, the analysis of high myopia and pathologic myopia (PM) remains challenging due to the need to process extensive datasets that incorporate both ocular imaging biomarkers and morphological alterations in the retina and choroid (20). **Table 1** lists some AI models for the diagnosis of high myopia and their advantage.

**Table 1:** Artificial intelligence (AI) models in the diagnosis of high myopia and complications.

Disease	Research	Model type	Modeling variables	Auc or accuracy	Advantage
myopic maculopathy	Takahiro S, et al (82).	DNN	SS-OCT	0.97	Classify OCT images without myopic macular lesions and OCT images with myopic macular lesions, such as mCNV and RS, with high accuracy
	Yonghao L, et al (83).	CNN	OCT macular images	0.961 to 0.999	1. Sensitivities equal to or even better than those of retina specialists, as well as high specificities. 2. Provided a transparent and interpretable diagnosis with heatmaps.
	Tien-En T, et al (84).	ResNet-101 (CNN)	CFP	0.969 or higher	The deep learning algorithms outperformed all six expert graders in the detection of each condition
	Juzhao Z, et al (85).	SSL	CFP	0.973 to 0.999	high accuracy and potential to enhance large-scale myopia screenings
Glaucoma	Ai-Su Y, et al (86).	VE-GCCLS and OMEGA-Net	SAP, PP, and OCT	0.887 ± 0.006	incorporate SAP and PP parameters; the highest diagnostic performance across all metrics
	An Ran R, et al (87).	a multi-task 3D-DL model	SD-OCT	0.949 and 0.965	The presence of MF did not affect the accuracy of GON detection
	Yen-Ying C, et al (88).	CBAM	CFP	0.894	Identify glaucoma by fundus photographs
	Christopher B, et al (8).	a deep learning autoencoder-based model	SS-OCT	0.92	With better diagnostic accuracy than the single autoencoder model, global RNFL thickness, and texture en face-based approaches.
	Swati S, et al (89).	a specialized ensemble network	OCT	0.92 ± 0.03	Structural variation in the ONH in H, HM, G, and HMG conditions
	Siamak Y, et al (90).	GEM	Visual field	–	Separated Frequency Doubling Technology (FDT) fields from healthy and glaucoma eyes and identified familiar glaucomatous patterns of loss.
	Nahida A, et al (91).	ResNet18 and VGG16	Humphrey 24–2 VF	97.00%	Similar to conventional global indices, and could assist clinicians in precision glaucoma detection and progression management
myopia onset and progression	Zengshuo W, et al (92).	a deep neural network (Myopic-Net)	CFP	87.30%	Identify the myopia onset and progression from fundus image pairs using anatomical changes in the optic disc and surrounding areas
	Jiajia L, et al (93).	EBM, GBDT, DNN, and XGBoost	modern behaviors and ocular biometrics, et al.	0.92; 71% accuracy	Adaptively integrating diverse datasets
	Min H, et al (94).	XGBoost	OCT and CFP	0.845 ± 0.050	Integrating multimodal data to enhance the accuracy of myopia onset prediction

### 5.1 AI in PM diagnosis

By training on extensive OCT image datasets, the convolutional neural network (CNN) model can accurately identify various ocular fundus lesions in patients with high myopia, such as retinal splitting, macular hole, retinal detachment, and pathological myopic choroidal neovascularization (83). The detection of these lesions achieves areas under the receiver operating characteristic curve (AUC) ranging from 0.961 to 0.999, with sensitivity surpassing that of retinal specialists and specificity consistently exceeding 90% (82). Furthermore, a deep learning algorithm based on retinal fundus photos also plays a significant role in diagnosing high myopia and its complications. By learning from large datasets of retinal images, these models extract key features to determine the presence of high myopia or pathologic myopia, as well as associated macular lesions (84). In a multi-cohort study, a deep learning system demonstrated robust diagnostic performance for myopic macular degeneration and high myopia. It achieved AUC values of 0.969 (95% CI: 0.959–0.977) or higher for myopic macular degeneration, and 0.913 (0.906–0.920) or higher for high myopia—outperforming six human experts in detecting each condition (84). Subsequently, a self-supervised learning-enhanced deep learning system was developed for identifying myopic maculopathy in highly myopic patients (85). External validation results attained 89.0% accuracy, 71.7% sensitivity, 87.8% specificity, and AUCs of 0.978 and 0.973. These findings underscore the potential of deep learning to enhance diagnostic accuracy in pathologic myopia and reduce dependence on human interpretation.

Continuous innovation has been observed in the development of artificial intelligence technologies for ophthalmic applications. A meta-analysis of ML methods in high myopia revealed that for diagnosing pathologic myopia, the summary receiver operating characteristic (SROC) was 0.97 (95% CI: 0.95–0.98), with sensitivity and specificity of 0.91 (95% CI: 0.89–0.92) and 0.95 (95% CI: 0.94–0.97), respectively (95). These high-performance AI systems show great potential for early detection—particularly in large-scale screening programs—by rapidly analyzing vast quantities of images and identifying individuals at high risk of high myopia or its complications.

To further improve diagnostic robustness, multimodal artificial intelligence systems have been developed that integrate diverse data sources, such as fundus photographs, OCT images, and clinical patient information, enabling a more comprehensive and accurate assessment (96). At the same time, increasing emphasis has been placed on model interpretability. Techniques such as gradient-weighted class activation mapping (Grad-CAM) are being utilized to visualize the AI decision-making process, thereby helping clinicians understand the basis of diagnoses and fostering greater trust in AI-generated results (97).

In large-scale screening initiatives, artificial intelligence has demonstrated considerable advantages. For example, in a community-based fundus disease screening study, an AI system used to analyze color fundus photographs exhibited high accuracy, specificity, and negative predictive value in detecting various retinal diseases—including pathologic myopia—providing a reliable method for identifying multiple retinal conditions in primary healthcare settings (98). These findings highlight AI’s ability to enhance both the efficiency and accuracy of diagnosing high myopia and pathologic myopia, thereby supporting early detection and timely intervention.

### 5.2 AI in myopic glaucoma diagnosis

Artificial intelligence has emerged as a powerful tool in the diagnosis of myopic glaucoma, serving as a crucial imaging modality in this context, and the integration of AI has significantly augmented its diagnostic potential. AI-enhanced OCT systems can analyze complex image data to detect early signs of myopic glaucoma with high precision (86). One key application lies in the measurement of retinal nerve fiber layer (RNFL) thickness. AI-driven analysis enables precise quantification of sectoral changes that may indicate early glaucomatous damage. For example, in highly myopic glaucoma (HMG) patients, the RNFL thinning rate at the temporal quadrant and eight o’clock sector was greater (99).

Deep learning models have been increasingly applied to analyze both OCT scans and fundus photographs for glaucoma detection. A multi-task three-dimensional (3D) deep learning model was trained to detect glaucomatous optic neuropathy (GON) and myopic features simultaneously from spectral-domain OCT volumetric scans (87). This model exhibited strong generalizability across different datasets, achieving an area under the receiver operating characteristic curve (AUROC) of 0.949 during internal validation for GON detection—surpassing the performance of average retinal nerve fiber layer (RNFL) thickness measurements.

However, most AI systems still rely on RNFL thickness for diagnosis, and more models are needed to enhance reliability, such as the en-face texture-based analysis method (88). The three-dimensional image system based on artificial intelligence may further enhance the diagnostic accuracy of glaucoma in people with high myopia (100). Because the structural changes induced by HM exist with more characteristics in three-dimensional space, such as the tilting of the optic disc and the traction of the sclera, this requires more data and further models to improve the diagnostic accuracy. Moreover, AI models trained on various types of eyes may perform better (8).

In addition to OCT-based approaches, AI systems have proven effective in analyzing fundus photographs. For example, a deep learning system trained on fundus photographs from a highly myopic population achieved an AUROC of 0.894 in identifying glaucoma cases (101). Further improving detection accuracy, researchers have employed a dual autoencoder model that integrates reconstruction errors from both healthy and glaucomatous training data, leading to enhanced performance in glaucoma identification (8). A recent study used a 3D deep learning approach to classify optic nerve head (ONH) structures by converting OCT scans into 3D point clouds and applying an ensemble network. This research successfully identified unique structural signatures of the ONH in glaucoma, high myopia, and their combination (HMG). The key differentiating features of HMG ONHs included focal cupping notch, deep optic cup excavation, thinning of the prelaminar tissue, and a significantly smaller MRW (89). This research supports the potential of morphological data in improving glaucoma diagnosis in myopic patients.

ML has also been applied to analyze visual fields in glaucoma. Unsupervised techniques, such as the Gaussian mixture model with expectation maximization (GEM), can distinguish abnormal visual fields (VFs) from normal ones and identify characteristic glaucomatous loss patterns (90). A CNN-based model developed for classifying VF images achieved high accuracy in separating glaucoma from normal cases (91). Furthermore, integrating functional VF parameters with structural OCT data through deep learning helps reduce diagnostic confounding caused by high myopia (86). Nevertheless, the application of AI in diagnosing glaucoma visual fields within myopic populations still demands further investigation.

High-performance AI systems can enhance large-scale screenings for myopic glaucoma, particularly in resource-limited settings. However, challenges such as dataset bias, limited external validation, and ethical concerns must be overcome before broader clinical implementation. In fundus photography-based diagnosis, AI-generated outcomes may influence clinical decisions—especially among less-experienced physicians (102). Despite these limitations, AI continues to hold promise for improving the accuracy and efficiency of glaucoma diagnosis in highly myopic patients.

### 5.3 Predictive models of myopia

Predicting the onset and progression of myopia plays a crucial role in the prevention and management of both high and common myopia, as it facilitates early detection of high-risk individuals, enables earlier diagnosis, and supports timely implementation of preventive strategies. Predictive models incorporate multiple factors to enhance accuracy. Age of myopia onset has been identified as an important predictor; for example, a study conducted among Singaporean children demonstrated that earlier onset of myopia was associated with an increased risk of developing high myopia later in childhood (103). This indicates the importance of early screening and monitoring in pediatric populations to identify those susceptible to HM and its complications.

Genetic factors also contribute substantially to prediction models. The identification of specific gene mutations linked to high myopia—such as variations in *LEPREL1*—can improve risk stratification, particularly in families with a history of myopia-related complications (104). Integrating genetic markers with clinical metrics holds promise for building more personalized and accurate predictive tools. Environmental influences represent another key dimension. Factors including limited time outdoors, prolonged near-work, and aspects of the living environment—such as high population density and constrained residential space—have been associated with myopia development (105). Combining environmental, genetic, and ocular parameters can lead to the development of more holistic and robust prediction models for high and pathological myopia.

Machine learning-based predictive models are being developed to assess the risk of complications in high myopia by considering demographic, genetic, environmental, and ophthalmic variables. For instance, a deep neural network named Myopic-Net demonstrates strong accuracy, reliability, and generalization in detecting and tracking myopia progression based on anatomical changes in retinal fundus images (92). Also, there are some studies explored the integration of multiple data to further improve the myopia progression prediction models. A merge model can integrate various risk factors, including ocular biometrics and genetic factors, and calculate a comprehensive risk score for each patient (93). However, different approaches for merging these models (sequential merging, simple averaging approach, and transfer learning) have variations in feature importance, which ultimately will affect the output results. Another mixed-model approach highlights the potential of multimodal data integration in enabling personalized strategies for myopia management. It integrated multimodal imaging—such as 3D optical coherence tomography (OCT) and color fundus photography (CFP)—and achieved an AUROC of 0.845 ± 0.050 for predicting myopia onset (94). Due to the numerous influencing factors of myopia and the limitations of existing models, it is challenging to create a truly integrated and personalized prediction model.

### 5.4 Limitations of AI

Although AI has been introduced to address the difficulties in diagnosing high myopia, there are still many unresolved challenges. First, the quality and quantity of data are of great significance for the application of artificial intelligence. However, the existing AI datasets usually only target one or a few specific groups of people. This may result in poor generalizability and makes it difficult to determine whether the poor performance is attributable to spectrum bias (106–108). Multicenter studies and public datasets can assist in addressing this issue. At present, there are several public datasets for other eye diseases, but none of them focus on myopia (109, 110).

The diagnosis made by AI is still based on the existing diagnostic criteria applied by physicians, and the training data includes the subjective judgments of the annotating doctors. Therefore, observer subjectivity introduces diagnostic variations. Reducing the subjectivity of human observers, AI can enhance the consistency of diagnoses (111). However, this improvement depends on the design and implementation of the AI system. Although AI can never exceed the scope of its training data, its good consistency and reproducibility can help reduce diagnostic discrepancies among different physicians, and even for the same physician at different time points. Furthermore, deep learning can extract complex feature combinations from data that are beyond direct human visual or cognitive perception, thereby helping to uncover objective indicators within the data. Hence, how to deploy AI more judiciously in diagnosis remains an important issue to be considered.

In addition, the “black box” model limits the understanding of both clinicians and patients, and the diagnostic decisions lack interpretability. The risks of “black box” algorithms are particularly critical as misdiagnoses can lead to irreversible vision loss. Using a model predicting glaucoma progression based solely on OCT-derived thickness maps, without understanding its reliance on peripapillary RNFL parameters, may cause clinicians to overlook confounding factors such as high myopia (112). Dataset biases can also propagate in black box systems, exacerbating diagnostic disparities (113). To reduce AI-related errors, the participation and supervision of human observers remain indispensable. Moreover, it is necessary to establish a clear framework to balance the supervisory duties of clinicians, the responsibilities of developers, and the management authority of the institution (114).

## 6 Future research directions

Future research on high myopia diagnostics should focus on several key areas to enhance detection and management. Advancements in imaging technology—such as higher-resolution optical coherence tomography (OCT), novel applications of OCT angiography, and emerging techniques like photoacoustic imaging—will be critical. Efforts may include improving OCT’s capacity to detect early scleral and choroidal changes, which remain poorly characterized in high myopia (115, 116).

Another priority is the discovery and validation of diagnostic biomarkers. Parameters such as choroidal thickness, retinal microvasculature, and genetic markers are under investigation. Initiatives like the China Alliance of Research in High Myopia (CHARM) project seek to quantify biomarkers—including fundus tessellation, optic nerve head features, and vascular metrics—using multimodal imaging and genetic data (7). These biomarkers could facilitate early diagnosis, predict progression, and guide personalized treatment.

Genetic research will continue to be essential. Identifying additional mutations associated with high myopia and elucidating their functional roles may reveal new pathological mechanisms and support genetic testing strategies.

Further development of artificial intelligence (AI) and machine learning (ML) is also needed. While AI has demonstrated potential in image analysis, future work should aim to refine algorithm generalizability across diverse populations and integrate AI tools into clinical workflows.
